# Integrative Analyses of Antler Cartilage Transcriptome and Proteome of Gansu Red Deer (*Cervus elaphus kansuensis*) at Different Growth Stages

**DOI:** 10.3390/ani12070934

**Published:** 2022-04-06

**Authors:** Yanxia Chen, Zhenxiang Zhang, Wenjie Jin, Zhaonan Li, Changhong Bao, Caixia He, Yuqin Guo, Changzhong Li

**Affiliations:** 1 College of Eco–Environment Engineering, Qinghai University, Xining 810016, China; kingsunny@126.com (W.J.); lizhaonan123456@163.com (Z.L.); bch656897@163.com (C.B.); hecaixia1119@163.com (C.H.); 2 Qinghai Academy of Animal Science and Veterinary Medicine, Qinghai University, Xining 810016, China; zhangzhenxiang6895@163.com; 3 Research Monitoring and Evaluation Center of Qinghai National Park, Xining 810016, China; guoyu_qg@126.com

**Keywords:** *Cervus elaphus kansuensis*, antler, endochondral ossification, rapid growth, histogenesis, multi-omics

## Abstract

**Simple Summary:**

Velvet antler is the only organ in mammals that can completely and circularly regenerate, which involves the co-development of a variety of tissues including cartilage. Thus, velvet antler can provide an ideal model for studying chondrogenesis, endochondral ossification and rapid tissue growth. However, the mechanism of rapid growth and regeneration of velvet antler is still unclear. In this study, we conducted integrated analysis of the transcriptome and proteome of antler cartilage tissues at different growth stages. The results showed that gene13546 and its coding protein rna13546 were annotated in Wnt signaling pathway. They may play roles in antler rapid growth and regeneration.

**Abstract:**

The velvet antler is a unique model for cancer and regeneration research due to its periodic regeneration and rapid growth. Antler growth is mainly triggered by the growth center located in its tip, which consists of velvet skin, mesenchyme and cartilage. Among them, cartilage accounts for most of the growth center. We performed an integrative analysis of the antler cartilage transcriptome and proteome at different antler growth stages. RNA-seq results revealed 24,778 unigenes, 19,243 known protein-coding genes, and 5535 new predicted genes. Of these, 2722 were detected with differential expression patterns among 30 d, 60 d, and 90 d libraries, and 488 differentially expressed genes (DEGs) were screened at 30 d vs. 60 d and 60 d vs. 90 d but not at 30 d vs. 90 d. Proteomic data identified 1361 known proteins and 179 predicted novel proteins. Comparative analyses showed 382 differentially expressed proteins (DEPs), of which 16 had differential expression levels at 30 d vs. 60 d and 60 d vs. 90 d but not at 30 d vs. 90 d. An integrated analysis conducted for DEGs and DEPs showed that gene13546 and its coding protein protein13546 annotated in the Wnt signaling pathway may possess important bio-logical functions in rapid antler growth. This study provides in-depth characterization of candidate genes and proteins, providing further insights into the molecular mechanisms controlling antler development.

## 1. Introduction

Gansu red deer (*Cervus elaphus kansuensis*) is commonly known as the white rump deer or Qilian red deer. It is an endemic species of the deer family in China and is mainly distributed in the forest areas of the Qilian Mountains. Research on *C. elaphus kansuensis* is limited with the focus on ecology, production performance, active substances, and functional gene cloning; however, omics studies are rare [1].

Deer antler is a unique cranial appendage derived from the forehead of male deer. It is attached to the skull by a tissue called the pedicle, from which the antler grows. Deer antler growth is relatively slow in the first month after the base shedding (30 d). Two months later (60 d), antler enters a rapid growth phase, which is the fastest growth rate of any mammal. At this stage, antler can grow up to 2 cm per day, and antler cells can reach more than 30 times faster than the proliferation of cancer cells, but no cancerous changes occur. At 90 days, the growth rate of velvet antler slows down and gradually ossifies, entering the ossification stage [2]. Histologically, deer antler is made up of several tissues such as velvet-like skin, cartilage, bone, nerves, and blood vessels [3]. Deer antleris the only mammalian organs that iscapable of annual regeneration and rapid growth [4]. Therefore, it is a premier model organ for cancer and regeneration research. The underlying molecular mechanisms involved in antler regeneration and rapid growth are the focus of attention of many biologists.

Advances in high-throughput technologies, including genomics, transcriptomics, proteomics, and metabolomics, have provided powerful methods to study antler development [5,6,7]. However, few studies have used an integrated multi-omics approach to characterize antlers in greater detail and comprehensively [8,9,10,11]. It is difficult to explain the biological regulatory networks of complex traits using only a single omics approach [12]. Integrative transcriptomics and proteomics analysis are more conducive to studying models of the regulatory mechanisms underlying the biological processes [13].

Transcriptomic and proteomic techniques have provided promising and powerful tools for mechanistic investigation as reflected in the increasing number of studies [13,14,15]. In addition, integrated transcriptomic and proteomic analyses comprehensively explain the influencing factors through experimentally observed differences in messenger ribonucleic acid (mRNA) and protein expression.

In this study, we combined mRNA sequencing with isobaric tags for relative and absolute quantitation (iTRAQ) to generate datasets encompassing primary (30 d), rapid (60 d), and ossified (90 d) stages of antler development. Our results provide a deeper understanding of rapid antler growth and cartilage development. To the best of our knowledge, this is the first study comprising genetic research on *C. elaphus kansuensis* the results of which provide a molecular basis for the further study of this species.

## 2. Materials and Methods

### 2.1. Animals, Sample Collection, and Preparation

In this study, *C. elaphus kansuensis* were fed on a semi-wild Shandan horse farm. Velvet antlers at different growth stages (30 d, 60 d, and 90 d) were collected from three adult male deer at each time point. Before we collecting antlers, the male deers were were anaesthetized with Mian Naining (Lu Mian ning, Beijing, China). After the velvet antlers were sawed off from the antler pedicle, potassium permanganate was used to stop the bleeding immediately. Then Lu xingling (Beijing, China) was injected to wake up red deers and release them into the wild. Cartilage parts in the antler tip were separated for transcriptome and proteome analyses and data verification. All samples were stored in liquid nitrogen tanks, brought back to the laboratory, and stored at −80 °C. All experimental protocols were approved by the Institutional Animal Care and Use Committee of Qinghai University (Xining, China), and all methods were carried out in accordance with approved guidelines and regulations (Code: SL-2022024).

### 2.2. RNA Extraction and Sequencing

Total RNA from the antler cartilage tissues of three Gansu red deer was extracted using The TRIzol reagent (Thermo Fisher Scientific, Waltham, MA, USA), and the extraction procedure was performed according to the manufacturer’s instructions. The extracted total RNA was quality checked using Agilent Bioanalyzer 2100 (Agilent, Santa Clara, CA, USA) and quantified using NanoDrop 2000 spectrophotometer (Thermo Fisher Scientific, Waltham, MA, USA). The RNA samples with RNA Integrity Number (RIN) ≥ 8 were selected for preparing libraries.

In total, 1 μg of RNA per sample was used as the input material for the RNA sample preparations. The NEBNext9 UltraTM RNA Library Preparation Kit for Illumina (NEB, USA) was used to conduct sequencing libraries according to the manufacturer’s procedures, and index codes were added to attribute sequences to each sample. Sequencing was performed using an Illumina HiSeq 2500 system (Illumina).

### 2.3. Protein Extraction, iTRAQ Labeling, and Liquid Chromatography-Electrospray Ionization-Tandem Mass Spectrometry Analysis

Total proteins of each sample were extracted using lysis buffer (7 M urea, 2 M thiourea, 2% CHAPS, proteasome inhibitor). The Qubit fluorescent protein quantification kit (Invitrogen) was used to quantify the protein concentration according to the manufacturer’s instructions. Protein peptides (100 μg) from each sample were labeled using the 8plex iTRAQ reagents multiplex kit (ABI, Foster City, CA, USA).

The iTRAQ-labeled samples were analyzed using a Q-Exactive mass spectrometer (Thermo Fisher Scientific) coupled with a nano high-performance liquid chromatography system (UltiMate 3000 LC Dionex; Thermo Fisher Scientific).

Peak recognition was performed on the peptide obtained by mass spectrometry, and a reference database was established. Then, Mascot 2.3.02 and Proteome Discoverer 1.4 (Thermo Fisher Scientific) were used for library search identification and quantitative analysis. Finally, the obtained peptides and proteins were analyzed using Uniport and NCBI databases, and their functions and metabolic pathways were annotated by Gene ontology (GO) annotation (*q* < 0.05) and Kyoto Encyclopedia of Genes and Genomes (KEGG) annotation (*q* < 0.05). The *p* value was adjusted using *q* value.

### 2.4. Transcriptome and Proteome Data Analysis

Transcriptome data were analyzed in accordance with RNA-Seq workflow (https://github.com/twbattaglia/RNAseq-workflow (accessed on 15 February 2017)). Raw data (raw reads, FASTQ files) from each library were preprocessed using in-house Perl scripts. Briefly, the adapters, sequences with unknown nucleotides larger than 10% (poly-N, N% > 10%) and low-quality reads (quality score < 20) were filtered from raw data. Concurrently, the sequences quality including Q20, Q30, GC content, were calculated. All downstream analyses were based on high-quality clean data. Hisat2 [16] was used to verify reads with a perfect match or one mismatch were further analyzed and annotated based on the reference genome of *C. elaphus* (https://www.ncbi.nlm.nih.gov/genome/?term=red+deer (accessed on 2 January 2018)). Then, clean reads were assembled and quantified using StringTie [17] to obtain contigs and unigenes. Differential expression analysis was performed using the EBSeq (default parameters, qtrm = 0.5) [18].

Significant differentially expressed genes (DEGs) among three groups were screened with the threshold of fold change ≥ 2 and false discovery rate (FDR) < 0.01 [19,20]. FDR was adjusted *p* value [7]. Only DEGs were subjected to further analyses.

For proteome analysis, the raw mass data were processed for peptide data analysis using Proteome Discoverer 1.4 (Thermo Fisher Scientific) with FDR = N(decoy) ∗ 2/(N(decoy)+ N(target) of < 1% and expected cutoff or ion score of < 0.05 with 95% confidence to search the Uniprot Human Complete Proteome database. Protein probabilities were assigned using the Protein Prophet algorithm [21], and proteins with at least two unique peptides were identified. Differential expression levels of proteins were calculated using the Mann–Whitney test and calibrated using the Benjamini–Hochberg correction. Only proteins with *p* value < 0.05 and fold-change ≥ 1.5 were defined as significant differentially expressed proteins (DEPs). The mass spectrometry proteomics data have been deposited to the ProteomeXchange Consortium via the PRIDE [22] partner repository.

### 2.5. Gene Functional Annotation of DEGs and DEPs

Based onClusters of Orthologous Genes (COG) [23], GO [24] and the KEGG database [25], the functions of DEGs and DEPs were annotated.

The Database of Clusters of Orthologous Genes (COGs) [23] is a Database of homeologous protein information maintained by NCBI. A protein can be compared to all the proteins in COGs and grouped into the appropriate COG cluster.

GOseq software was used for GO enrichment analysis of differential genes based on the Wallenius noncentral hypergeometric distribution mathematical model [26]. GO terms were considered to be significantly enriched with *q* < 0.05.

KEGG [25] is a database that integrates genomic, chemical, and phylogenetic information. The core of them are KEGG PATHWAY and KEGG ORTHOLOGY databases. In the KEGG PATHWAY database, biological metabolic pathways are classified into six categories of processing: cellular, environmental information, genetic information, human diseases, metabolism, and organismal systems. We used KOBAS software [27] to test the statistical enrichment of DEGs and DEPs in the KEGG pathways. KEGG pathways were considered to be significantly enriched with *q* < 0.05

### 2.6. Integrated Analysis of Transcriptomic and Proteomic Data

Integrated analysis was conducted to explore the consistency between proteome and transcriptome levels of antler cartilage at 30 d, 60 d, and 90 d. Spearman’s correlation test [28] was used to assess the correlation between gene expression levels in the transcriptome and corresponding proteins in the proteome. The results were divided into three categories: DEGs and DEPs had the same expression trend, DEGs and DEPs had reverse expression trend, and DEGs and DEPs had no expression difference.

## 3. Results

### 3.1. Data Availability

Raw FASTQ files of the transcriptome were submitted to the National Center for Biotechnology Information Sequence Read Archive (NCBI SRA) database under the BioProject Accession Number PRJNA772802 [29](Transcriptome profiles of velvet antler in Gansu red deer. Available online: http://www.ncbi.nlm.nih.gov/bioproject/772802 (accessed on 19 October 2021)). Proteomic data have not been submitted. The mass spectrometry proteomics data have been deposited to the ProteomeXchange Consortium (http://www.proteomexchange.org (accessed on 19 October 2021)) with the dataset identifier PXD032668.

### 3.2. RNA Sequencing and Assembly

We performed RNA-seq to investigate the mRNA expression profiles of cartilage in antler tips of *C. elaphus kansuensis* at different growth stages. After sequencing and filtration, we generated 50,552,622, 69,330,832, and 44,833,400 clean reads of the antler growth center for 30 d, 60 d, and 90 d stages, respectively. We obtained 40,317,002, 56,820,843, and 35,010,141 mapped reads after alignment to the reference genome, accounting for 79.75%, 81.96%, and 78.09% of clean reads from the three libraries, respectively. After mapping the read assembly using StringTie software, we obtained 24,778 unigenes. The lengths of the assembled unigenes ranged from 90 bp to 82,366 bp, with an average of 2269 bp and N50 of 3894 bp. Of these 24,778 unigenes, 19,243 were mapped to known protein-coding genes and 5535 were mapped to new predicted genes. After applying the fragments per kilobase of transcript per million mapped reads (FPKM) filter (FPKM = 0), 18,707, 19,694, and 18,985 unigenes were detected in 30 d, 60 d, and 90 d libraries, respectively. Among these genes, 17,527, 18,307, and 18,004 were detected (FPKM ≥ 0.1), whereas the remaining 7251, 6471, and 6774 genes were considered to be very low in expression or not expressed (FPKM < 0.1) in the three libraries.

Expression analysis showed that gene4406 and gene13363 were the most highly expressed in all three libraries (Table 1). The co-expressed gene7568, gene2851, and gene21360 also had higher expression levels. Among the most highly expressed genes, most were known genes; hence, only one and three predicted novel genes were selected in the 30 d and 90 d antler growth centers, respectively.

### 3.3. Differentially Expressed Genes

**Figure 1:**
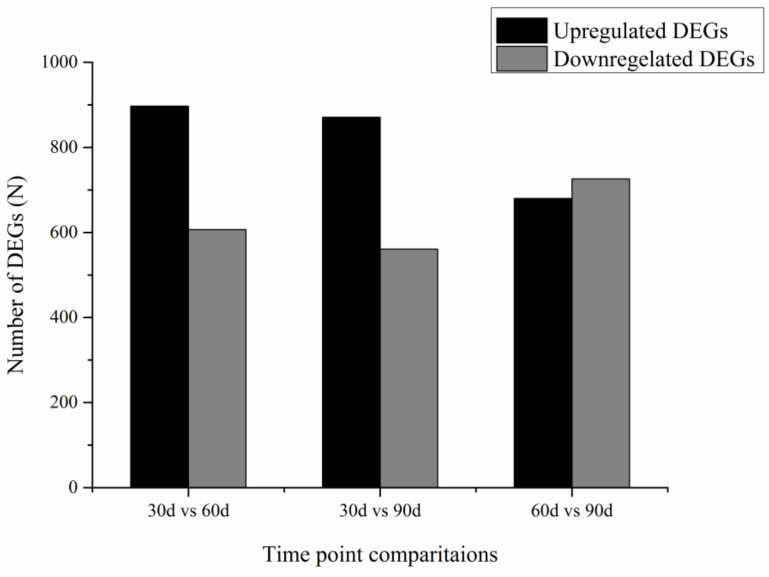
Statistical histogram of upregulated DEGs and downregulated DEGs in the three libraries. DEGs in the cartilage tissue of the velvet antler at the three time points were analyzed. There were more upregulated DEGs than downregulated DEGs at 30 d vs. 60 d and 30 d vs. 90 d, whereas there were fewer upregulated DEGs than down-regulated DEGs at 60 d vs. 90 d.

**Figure 2:**
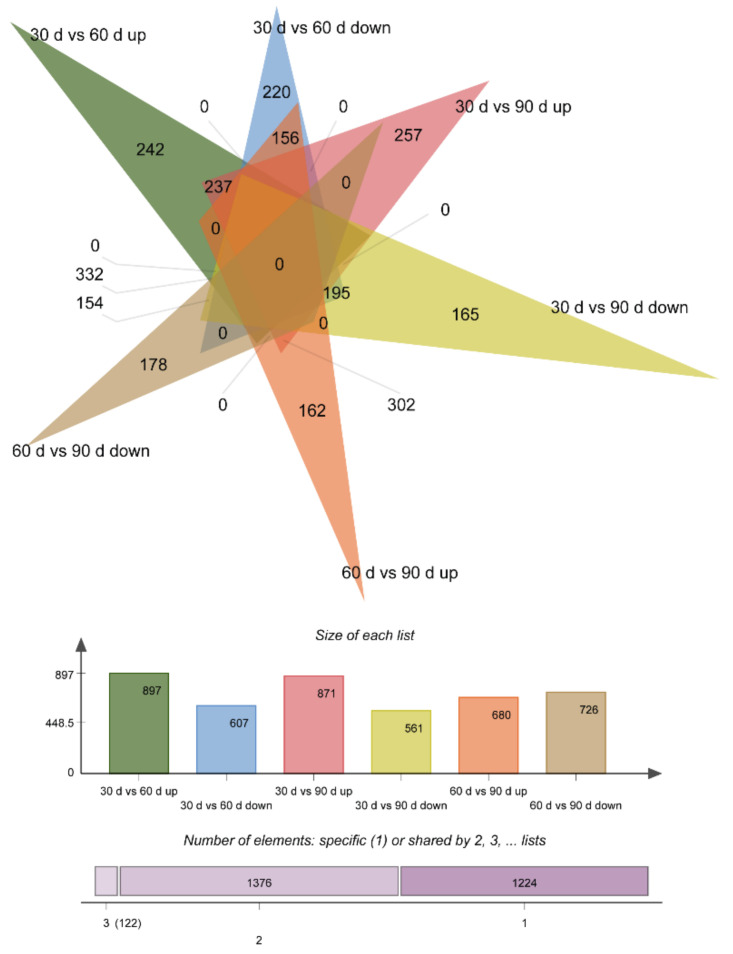
Venn diagram of differentially expressed genes. In the Venn diagram, each triangle represents the differential genes in a comparison combination (30 d vs. 60 d, 30 d vs. 90 d, and 60 d vs. 90 d), the number of overlapping triangular regions represents the number of common differential genes among the corresponding comparison combinations, and the non-overlapping region represents the unique differential genes in each comparison combination. A histogram corresponding to the Venn diagram can identify upregulated and downregulated genetic information for comparison combinations. Venn results showed that 30 d vs. 60 d had the maximum number of DEGs (1504), and 30 d vs. 90 d (1432) and 60 d vs. 90 d (1406) had the lowest number of DEGs.

Gene expression levels were calculated according to FPKM arithmetic; FDR < 0.01 and |log2 (foldchange)| ≥ 2 were set as the threshold for significantly differential expression. Finally, we generated 2722 differentially expressed genes (DEGs) among 30 d, 60 d, and 90 d libraries. Of these, 122 DEGs were co-expressed in the three libraries, 1504 DEGs in 30 d vs. 60 d (897 upregulated and 607 downregulated), 1432 DEGs in 30 d vs. 90 d (871 upregulated and 561 downregulated), and 1406 DEGs in 60 d vs. 90 d (680 upregulated and 726 downregulated) (Figure 1 and Figure 2).

### 3.4. Functional Annotation of DEGs

**Figure 3:**
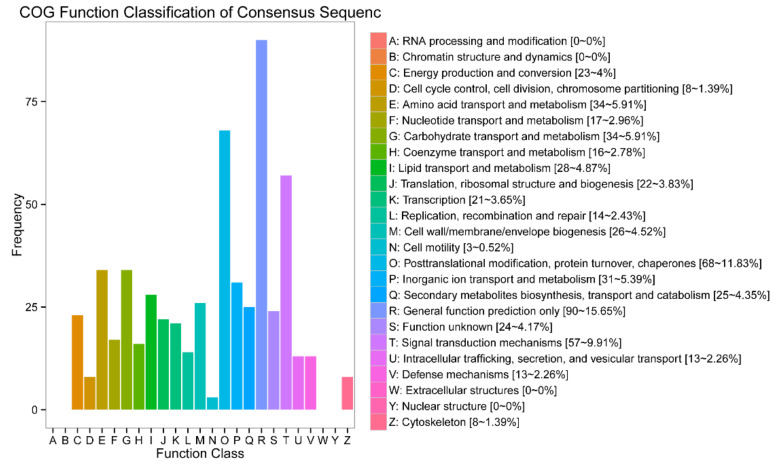
COG classification of all DEGs. COG classification is an important aspect of the functional annotation of DEGs. Among all COG terms, “R: General function prediction only” (90 DEGs, 15.65%), “O: Posttranslational modification, protein tumor, chaperones” (68 DEGs, 11.83%), and “T: Signal transduction mechanisms” (57 DEGs, 9.91%) classified the most DEGs.

Basic Local Aalignment Search Tool (BLAST) [30] software was used to align DEGs to COG [23], GO [24], and KEGG [25] databases using an E value cut-off of 10^−5^. The results indicated that among 2722 DEGs, 2290 were annotated against the GO database, 1705 against the KEGG database, and 529 against the COG database. Annotation of DEGs showed that 529 DEGs were predicted and classified into 25 functional categories after COG annotation (Figure 3). Most DEGs were classified into “R: General function prediction only,” followed by “O: Posttranslational modification, protein turnover, chaperones” and “T: Signal transduction mechanisms”.

**Figure 4:**
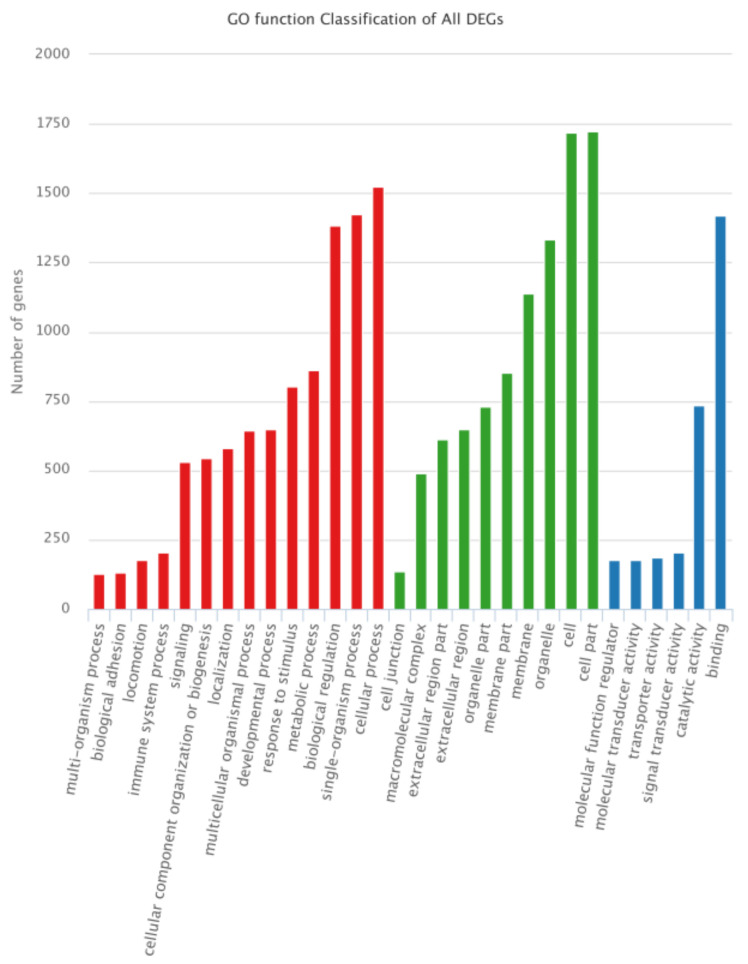
GO classification of all DEGs. For GO annotation, the abscissa is the GO term, and the ordinate indicates the number of DEGs annotated to GO terms.

In the GO annotation (Figure 4), “cellular process” (1525 DEGs), “single-organism process” (1425 DEGs), and “biological regulation” (1385 DEGs) were dominant in the biological process category; “cell part” (1722 DEGs), “cell” (1718 DEGs), “organelle” (1335 DEGs), and “membrane” (1139 DEGs) were dominant in the cellular component category, and “binding” (1418 DEGs) and “catalytic activity” (736 DEGs) were dominant in the molecular function category.

According to the KEGG annotation results (Supplementary Figures S1), the most classifications include “Pathway in cancer” (82 DEGs), “P13K-Akt signaling pathway” (76 DEGs), “Focal adhesion” (54 DEGs), “Rap1 signaling pathway” (53 DEGs), “HTLV-I infection” (51 DEGs), “Protein digestion and absorption” (51 DEGs), “Axon guidance” (51 DEGs), and “Proteoglycans in cancer” (50 DEGs).

**Figure 5:**
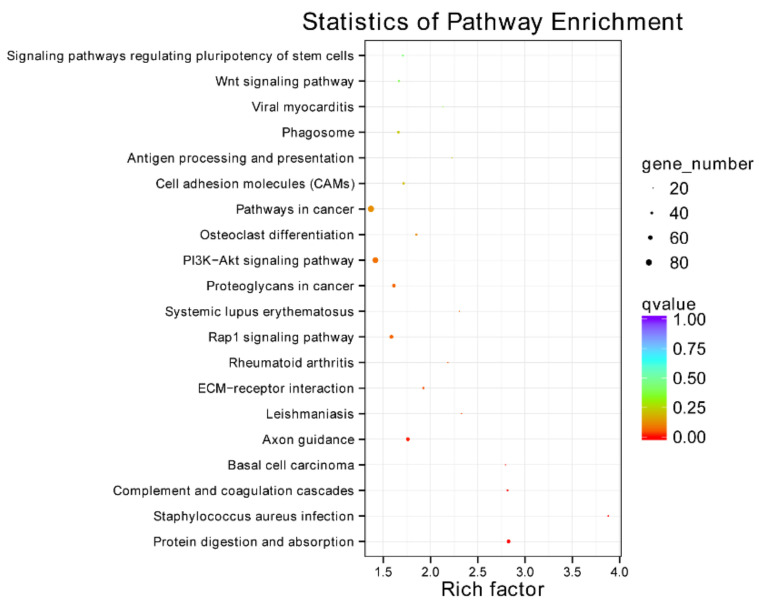
KEGG classification of all DEGs. For KEGG annotation, each circle represents a pathway, the ordinate indicates the name of the pathway, and the abscissa is the enrichment factor. The color of the circle represents the *q* value, which is the *p* value after multiple hypothesis test correction. The circle size indicates the number of target genes enriched in the pathway. Pathways with redder colors and larger circles had greater reference values. Twenty pathways with minimum q values are displayed in this study.

The KEGG analysis showed that DEGs were mainly associated with the “Pathways in cancer” and “PI3K-Akt signaling pathway”, followed by pathways of “Proteoglycans in cancer”, “Rap 1 signaling pathway”, “Axon guidance” and “Protein digestion and absorption” (Figure 5).

In addition, GO and KEGG annotations for DEGs in the comparisons of 30 d vs. 60 d, 30 d vs. 90 d, and 60 d vs. 90 d were also performed (Supplementary Figures S2 and S3).

### 3.5. Antler Proteins Revealed by iTRAQ Analysis

Proteome profiles of deer antlers were obtained by using the iTRAQ method. In total, 1540 proteins were identified, and all these proteins were expressed in 30 d, 60 d, and 90 d libraries (Table 2). Of these predicted proteins, 1361 were partially sequenced similar to known proteins, and 179 proteins were predicted as novel proteins.

### 3.6. Differential Expression of Proteins

**Figure 6:**
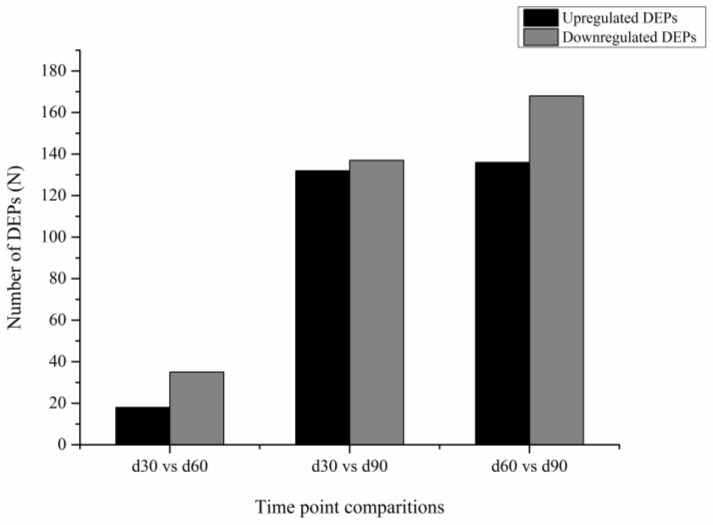
Statistical histogram of DEPs in three libraries. The DEPs in the cartilage tissue of the velvet antler at three time points were analyzed. There were more downregulated DEPs than upregulated DEPs in the 30 d vs. 60 d, 30 d vs. 90 d, and 60 d vs. 90 d comparisons.

**Figure 7:**
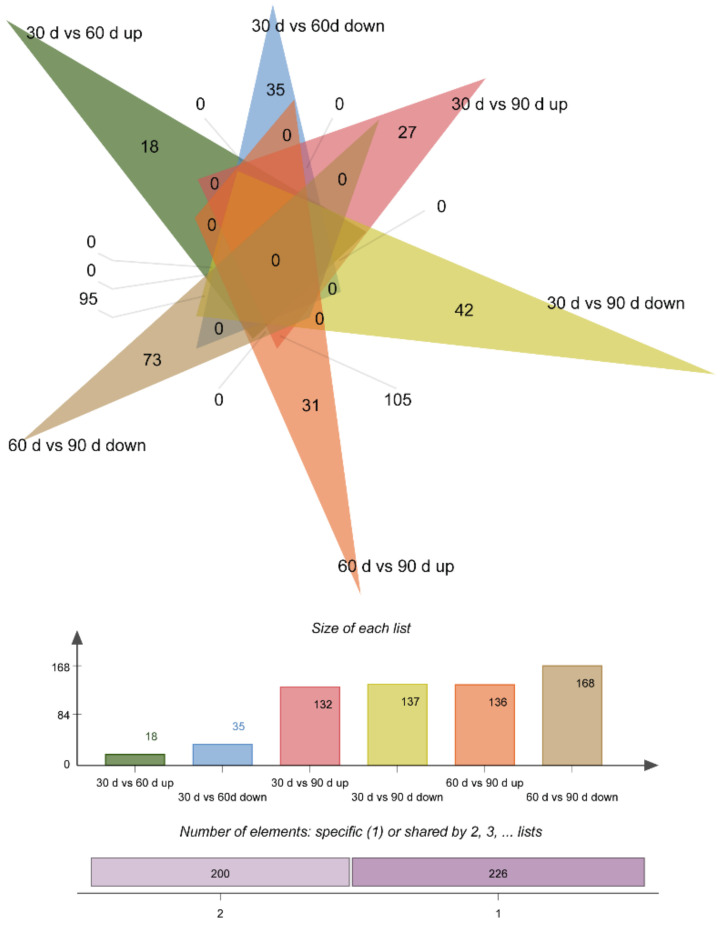
Venn diagram of DEPs. In the Venn diagram, each triangle represents the differential proteins in a comparison combination (30 d vs. 60 d, 30 d vs. 90 d, and 60 d vs. 90 d), the number of overlapping triangular regions represents the number of common differential genes among the corresponding comparison combinations, and the non-overlapping region represents the unique differential genes in each comparison. A histogram corresponding to the Venn diagram can identify upregulated and downregulated DEPs in each comparison. Venn results showed that 60 d vs. 90 d had the maximum number of DEPs (304), and 30 d vs. 90 d (269) and 30 d vs. 60 d had the lowest number of DEPs (53).

In total, 382 DEPs were screened in the three comparative analyses, of which 53 DEPs were expressed in 30 d vs. 60 d (18 upregulated and 35 downregulated), 269 DEPs in 30 d vs. 90 d (132 upregulated and 137 downregulated), and 304 DEPs in 60 d vs. 90 d (136 upregulated and 168 downregulated) (Figure 6 and Figure 7).

### 3.7. Annotation of DEPs

**Figure 8:**
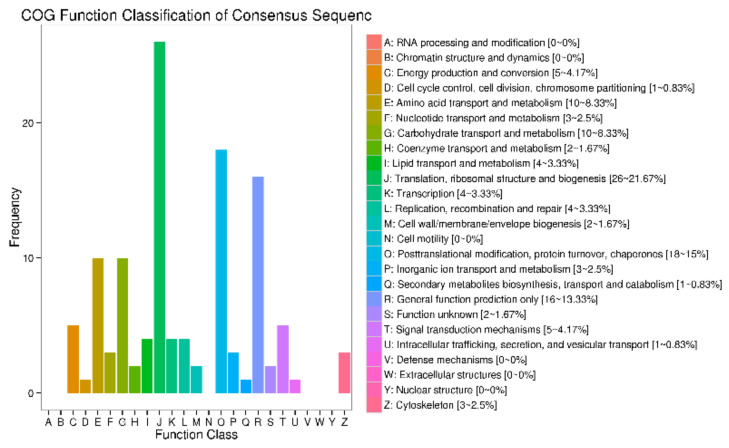
COG classification of all DEPs. Among all COG terms, “J: Translation, ribosomal structure, and biogenesis” (26 DEPs, 21.67%), “O: Posttranslational modi-fication, protein tumor, chaperones” (18 DEPs, 15%), and “R: General function pre-diction only” (16 DEPs, 13%) classified the most DEGs. The COG results of DEPs showed some differences compared with those of DEGs.

Based on the COG annotation, DEPs were classified into 19 functional categories (Figure 8). “Translation, ribosomal structure, and biogenesis” was the most popular group, followed by “Posttranslational modification, protein turnover, chaperones” and “General function prediction only.”

**Figure 9:**
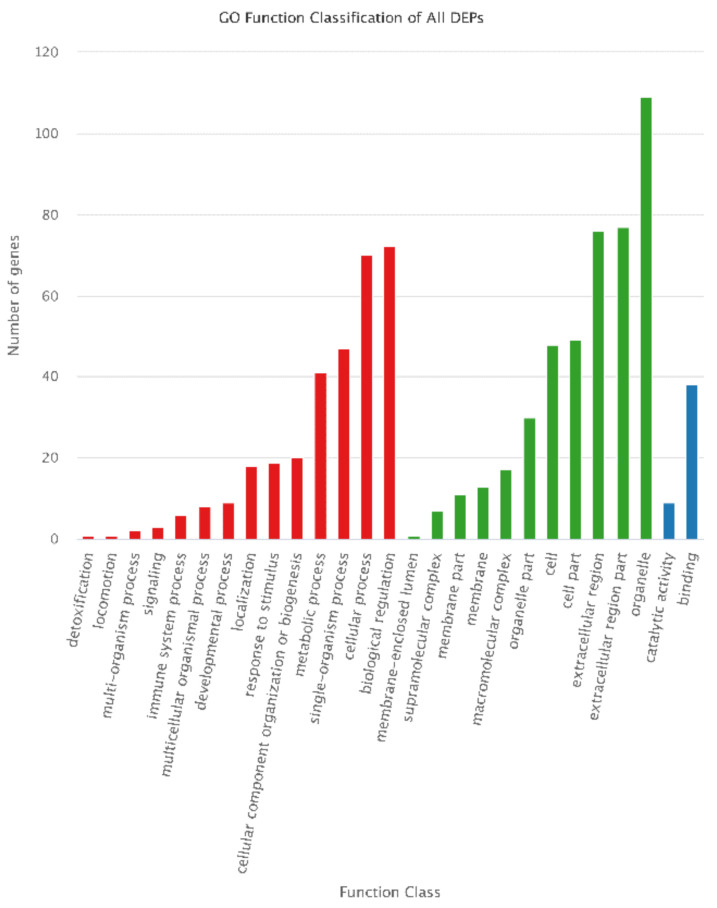
GO classification of all DEPs. For GO annotation, the abscissa is the GO term, and the ordinate indicates the number of DEGs annotated to GO terms.

In the GO annotation, “biological regulation” and “cellular process” were dominant in biological process’s category. In cellular component category, “organelle,” “extracellular region part,” and “extracellular region” were dominant. Furthermore, “Binding” was dominant in molecular function category (Figure 9). The results were different from those of DEGs.

**Figure 10:**
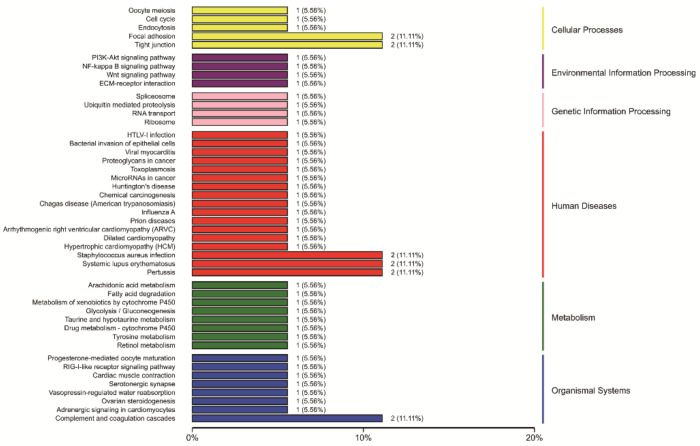
KEGG classification of all DEPs. For KEGG annotation, the ordinate in-dicates the name of the pathways, and the abscissa is the number and percentage of DEPs classified into the pathways. Owing to the small number of screened DEPs, few DEPs were annotated to each signaling pathway.

According to the KEGG annotation results (Figure 10), DEPs were annotated and assigned to 46 KEGG pathways. The pathways of “Tight junction,” “Focal adhesion,” “Pertussis,” “Staphylococcus aureus infection,” “Systemic lupus erythematosus,” and “Complement and coagulation cascades” classified two DEPs. In addition, other pathways only classified one DEP.

GO and KEGG annotations of DEPs at 30 d vs. 60 d, 30 d vs. 90 d, and 60 d vs. 90 d were also performed (Supplementary Figures S3 and S4).

In addition, we found 34 DEGs and 5 DEPs classified into the “Wnt signaling pathway,” and 82 DEGs and 7 DEPs classified into “Pathway in cancer.” These DEGs and DEPs require further study.

The rapid growth period (60 d) is a special physiological period in the growth process of the velvet antler, during which the growth rate of velvet antler cells was much higher than that of cancer cells. Moreover, the ordered tissue structure is still maintained without cancerization, and complete ossification eventually occurs, resulting in the death of velvet antler tissues and effectively preventing the growth of velvet without restriction. As velvet antler cell proliferation can be stopped eventually, the relationship between the growth and development mechanism of velvet antler and the malignant proliferation of cancer cells has been an attractive research topic. Furthermore, the velvet antler growth mechanism can be used to identify new ideas for cancer treatment. Thus, in the present study, we focused mainly on the DEGs and DEPs that had differential expression levels at 30 d vs. 60 d and 60 d vs. 90 d but had no differential expression at 30 d vs. 90 d. Finally, 488 DEGs and 16 DEPs with these characteristics were selected.

**Figure 11:**
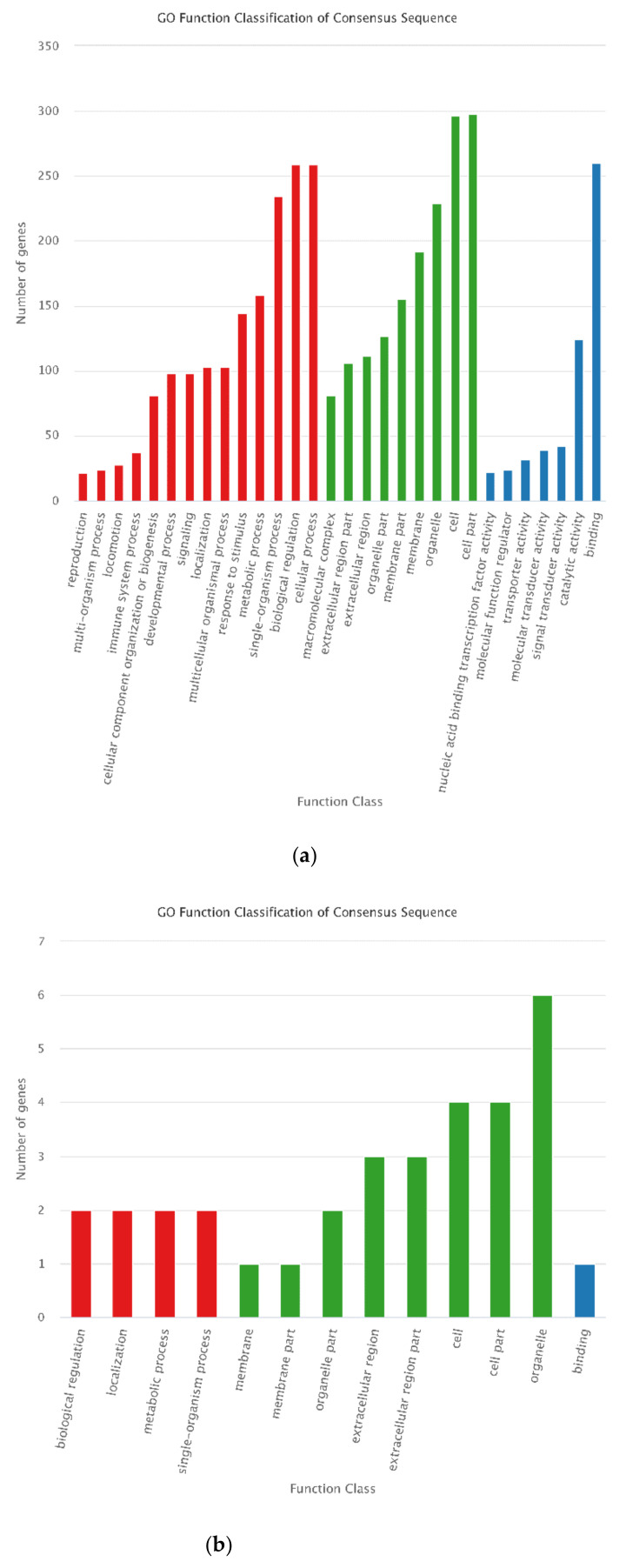
GO classification of the 488 DEGs and 16 DEPs. For GO annotation, the abscissa is the GO term, and the ordinate indicates the number of DEGs annotated to GO terms. (**a**) GO classification of the 488 DEGs. (**b**) GO classification of the 16 se-lected DEPs.

**Figure 12:**
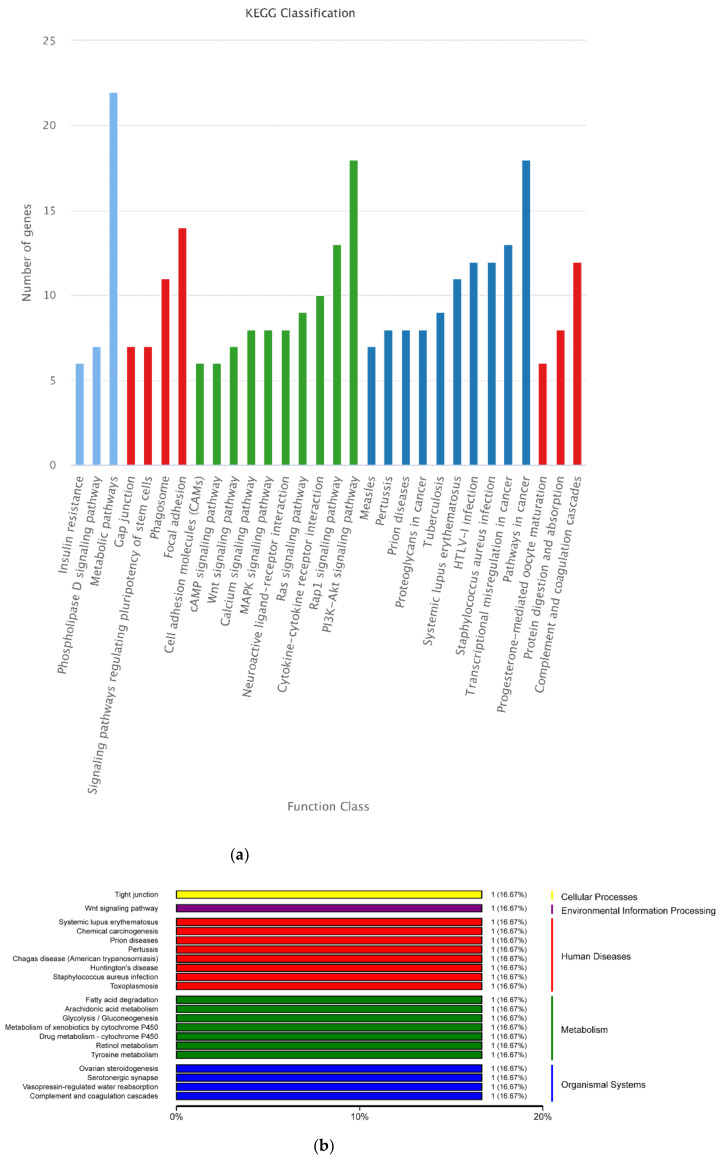
KEGG classification of the 488 DEGs and 16 DEPs. (**a**) KEGG classifica-tion of the 488 DEGs. “Metabolic pathways,” “PI3K-Akt signaling pathway,” and “Pathways in cancer” classified the most DEGs. (**b**) KEGG classification of 16 DEPs. Only one DEP annotated to each pathway.

To comprehensively understand the functions of the selected DEGs and DEPs, GO and KEGG functional enrichment analysis was conducted for the 488 DEGs and 16 DEPs. “cellular process” and “biological regulation” in biological process category, “cell” and “cell part” in cellular component category, and “binding” in molecular function category were the most enriched GO terms for the 488 DEGs. For the 16 DEPs, “organelle” classified most proteins (Figure 11). KEGG analysis showed that most of the selected DEGs were annotated in “metabolic pathway,” and then in “PI3K-Akt signaling pathway” and “pathways in cancer.” However, all KEGG pathways classified only one of the selected DEPs (Figure 12).

### 3.8. Integrated Analysis of Transcriptome and Proteome Data and Integrated Analysis of DEPs and DEG during Antler Growth

Integrated analysis of DEGs and DEPs during antler growth was performed. For integrated analysis, if a gene was detected to be expressed at the mRNA and protein levels simultaneously, there is a correlation between the mRNA and protein of the gene. The relationships between the number of proteins and genes were shown in Table 3.

Compared datasets of proteomics and transcriptomics showed that in 30 d vs. 60 d comparison, 108 proteins conjoined with their corresponding mRNAs. Among them, 76 NDEPs (Non-differentially expressed proteins) conjoined with DEGs, 24 DEPs conjoined with NDEPs, while 8 DEPs conjoined with DEGs. The eight identified DEGs/DEPs had reversed expression trends, which meant that the eight genes collectively exhibited up- or down-regulated expression profiles at mRNA and protein levels. In the 30 d vs. 90 d comparison, 314 proteins or transcripts were identified. Among them, 76 NDEPs conjoined with DEGs, 213 DEPs conjoined with NDEPs, and 25 DEPs conjoined with DEGs, of which 9 DEPs and DEGs had the same expression trend and 16 DEPs and DEGs had the reverse expression trend. In addition, 382 proteins or transcripts were identified in the 60 d vs. 90 d comparison. Among them, 80 NDEPs conjoined with DEGs, 257 DEPs conjoined with NDEPs, and 45 DEPs conjoined with DEGs, of which 16 DEPs and DEGs had the same expression trend and 29 DEPs and DEGs had the reverse expression tend.

In the three comparison groups, the number of conjoined DEPs/DEGs was lower than that of DEPs/NDEGs and NDEPs/DEGs. Therefore, integrative DEGs and DEPs were used for further analyses.

**Figure 13:**
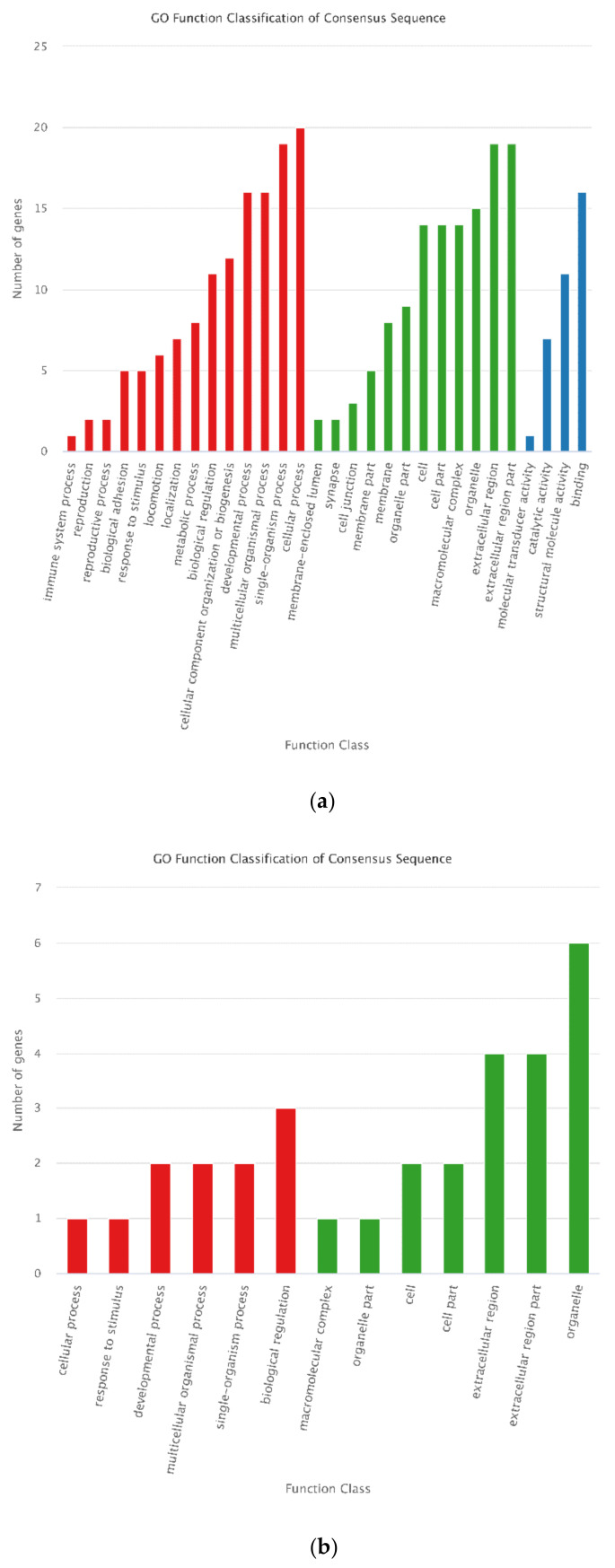
GO classification of DEGs and DEPs with the same trend. For GO anno-tation, the abscissa is the GO term, and the ordinate indicates the number of DEGs annotated to GO terms. (**a**) GO classification of DEGs with the same trends to DEPs. (**b**) GO classification of DEPs with the same trends to DEGs.

We performed GO classification for DEPs and their corresponding DEGs with the same expression trends in the three libraries. In total, 22 DEPs and 12 DEGs were selected after filtering the repeat ID. Then, these DEPs and DEGs were classified into GO terms (Figure 13). The term “cellular process” classified the most DEGs but “organelle” classified the most DEPs. All terms annotated by DEPs were also annotated by DEGs, but some terms were annotated only by DEGs.

**Figure 14:**
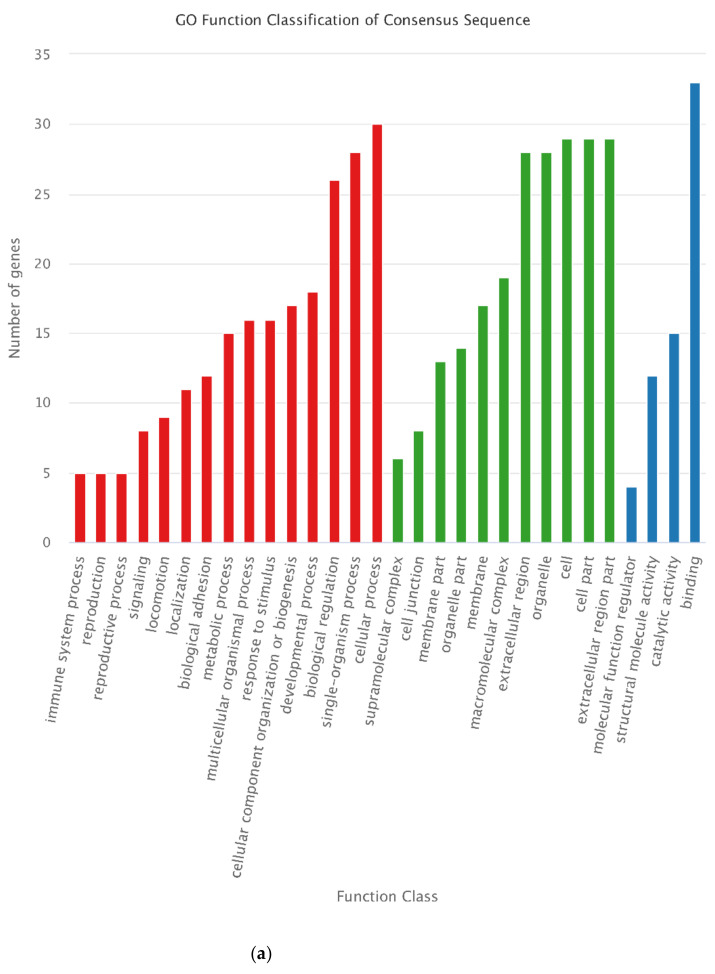

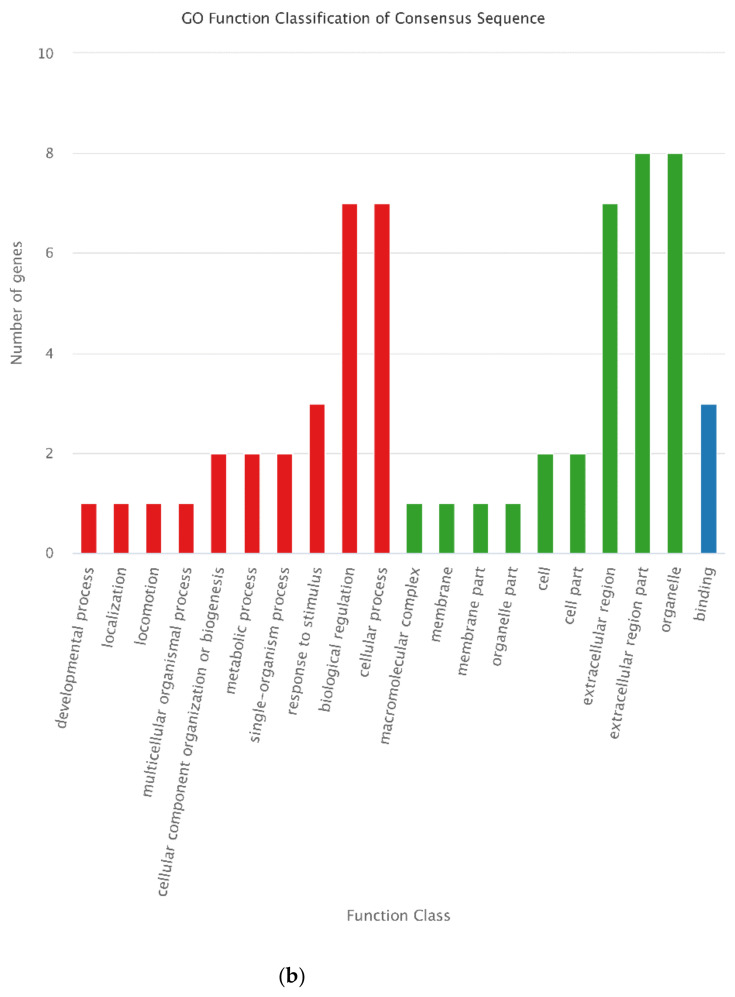
GO classification of DEGs and DEPs with the reverse trend. For GO an-notation, the abscissa is the GO term, and the ordinate indicates the number of DEGs annotated to GO terms. (**a**) GO classification of DEGs with the reverse trend to DEPs. (**b**) GO classification of DEPs with the reverse trend to DEGs.

Then, GO classification for DEPs and their corresponding DEGs with the reverse expression trend in the three libraries was conducted; we screened 40 DEPs and 29 DEGs. For GO classification (Figure 14), the term “binding” classified the most DEGs but “extracellular region part” and “organelle” classified the most DEPs. Similar to the DEGs and DEPs, DEGs also annotated all terms annotated by DEPs, but some terms were only annotated by DEGs.

**Figure 15:**
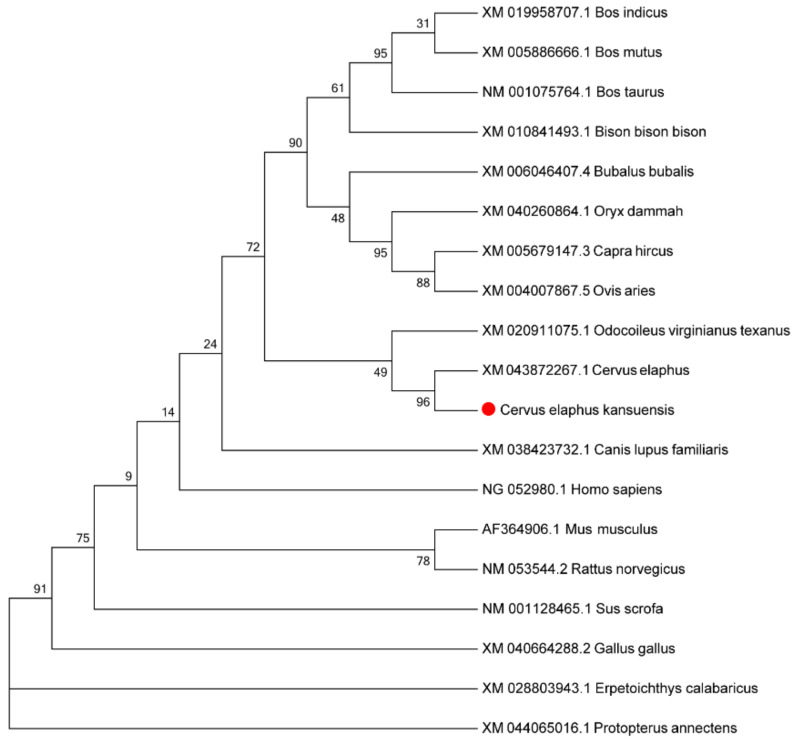
Phylogenetic tree analysis of *C. elaphus kansuensis* SFRP4 with other species. The tree was constructed using a matrix based on protein sequences and the program MEGA 7 [34].

Among the 488 DEGs, 2 genes (gene13546 and gene6151) encode proteins (protein13546 and protein6151) in the 16 selected DEPs. Gene13546 (protein13546) was annotated in “Wnt signaling pathway” and gene6151 (protein6151) was annotated in “complement and coagulation cascades,” “Prion diseases, Pertussis,” “Chagas disease (American trypanosomiasis),” “Staphylococcus aureus infection,” and “Systemic lupus erythematosus” KEGG pathways. It has been reported that the Wnt signaling pathway participates in antler development [31]. Thus, gene13546 and its encoding protein (protein13546) may play important roles in rapid antler growth. Unfortunately, owing to the incomplete genome of red deer, detailed gene names were not annotated in the transcriptome data. However, BLAST results showed that gene13546 was similar to the SFRP4 gene in other mammals and humans, which could be predicted as the SFRP4 gene in Gansu red deer. A phylogenetic tree was constructed on SFRP4 amino acid sequence of *C. elaphus kansuensis* and 18 other species including *Cervus elaphus* and *Bos tauus* (Figure 15). The results showed that *C. elaphus kansuensis* was closest to Cervidae, followed by bovidae, and furthest to the fish. This was similar to the results of previous studies [32,33].

## 4. Discussion

Deer antler cells are normal, non-cancerous cells but can proliferate and differentiate rapidly. This property makes the antler a valuable model for studying potent growth factors, unique signaling pathways, and novel regulatory systems [9]. The growth center of the antler tip determines the rapid growth rate of the antler and is precisely regulated so that the antler does not become cancerous [35]. The antler tip region is referred to as the proliferation zone [36]. The rapid growth of antlers is mainly achieved through the activity of cells residing in the proliferation zone [37]. Therefore, the apical tissue is often used as the research object to study the rapid growth and endochondral ossification mechanisms of deer antlers. Presently, the molecular mechanism of antler regeneration and rapid growth needs further study.

### 4.1. General Features of the Transcriptomes and Proteomes of Antlers at Different Growth Stages

In this study, transcriptomic and proteomic analyses were performed on the antler cartilage tissue of Gansu red deer grown at 30 d, 60 d, and 90 d, and DEGs and DEPs were screened at different growth stages. For RNA-seq, clean reads as a percentage of total raw reads, Q30 values, and GC content are often used to assess the quality of transcriptome sequencing [4]. The Q30 values were 94.50%, 95.31%, and 94.15% at 30 d, 60 d, and 90 d antler cartilage tissues, respectively. The GC content was 56.66%, 55.32%, and 55.37% in the three samples, respectively. These data indicate that the transcriptome sequencing quality was qualified for subsequent analysis in this study. After assembly, we obtained a total of 24,778 unigenes, with an average of 2269 bp and an N50 of 3894 bp, which is longer than the previous transcriptome studies of deer antler [38,39]. Proteins expressed in antlers at different growth stages were also identified by the iTRAQ method in this study. There were previous studies have reported differentially expressed proteins involved in antler regeneration [8,11]. How our data differ from the reported findings has not been analyzed. Proteins that regulate rapid antler growth and chondrogenesis require further exploration.

In this study, 3 male red deer were selected to collect antler samples at about 30 d, 60 d and 90 d growth stages, so that there were 3 samples from different individuals at each time point. In high-throughput sequencing, every sample pool was a mix of equal amount of RNAs from three individual male deer, as was the case in other studies [7,40,41,42], which indicated that this method was acceptable. However, studies with at least biological replicates may be more meaningful and acceptable compared with RNA pools, though each pool is collected with at least three samples. To explore the moleculars involved in rapid antler growth, integrative analysis of transcriptomic and proteomic data was meaningful, which helped us to screen a set of genes and proteins that may be related to antler rapid growth.

### 4.2. Analysis of Expression of Key DEGs and DEPs Involved in Rapid Antler Growth

High neuralization and vascularization are important tissue characteristics of the velvet antler [43]. The velvet antler enters the rapid growth stage from the beginning to about 60 days [44]. At the rapid growth stage, the growth rate of the velvet antler reaches 2 cm/d. Many scholars call this phenomenon cancer-like growth [45,46]. The behavior of the velvet antler is very similar to the expansion of cancer cells, but the fascination is that it does not become cancerous. During the ossification period, the growth rate begins to slow down again. This unique growth process makes the rapid growth and ossification mechanism of the velvet antler a major focus of biological research. In our study, comparative transcriptome analysis showed that DEGs in comparisons of 30 d vs. 60 d, 30 d vs. 90 d, and 60 d vs. 90 d were mainly classified in “pathways in cancer” and “PI3K-Akt signaling pathway.” This was consistent with the enrichment of DEGs. As antler growth is a tumor-like development, the genes classified in pathways in cancer may be potential regulators in antler development. The PI3K-Akt signaling pathway can be activated by a variety of factors and has a wide range of biological functions, such as transcription, translation, cell cycle, proliferation, differentiation, survival, apoptosis, metabolism, angiogenesis, and migration [47,48]. It has been implicated in various cancers, such as gynecological tumors [49], prostate cancer [50], medulloblastoma [51], and gastric cancer [52]. In addition, the PI3K-Akt signaling pathway participates in chondrocyte proliferation, apoptosis, autophagy [53,54], cartilage protection [55,56], cartilage degeneration [57], cartilage cell apoptosis [58], and osteoarthritis cartilage regeneration [59]. Liu [60] found that the PI3K/Akt pathway affects the generation and regeneration of antler stem cells in vitro. Dong [61] predicted that the PI3K-Akt signaling pathway plays an important role in regulating the regeneration of antlers. Furthermore, proteomic analysis of velvet cartilage showed that DEPs were classified in the “PI3K-Akt signaling pathway” and “Wnt signaling pathway.” Thus, the genes and proteins involved in the development of cartilage tissue in the growth center of deer antlers at the fast growth stage require further study.

Integrated analysis showed that among the 488 DEGs and 16 DEPs, 2 genes (gene13546 and gene6151) and proteins (protein13546 and protein6151) corresponded to each other. Gene13546 and its encoding protein protein13546 were both annotated in the “Wnt signaling pathway.” Wnt signaling plays a crucial role in embryogenesis [62]. In adults, Wnt is mainly involved in cell proliferation and differentiation [62]. Clinical findings suggest that Wnt signaling is critical for trabecular and cortical bone mass [63]. The Wnt signaling pathway has been reported to regulate antler regeneration [31,64]. Secreted frizzled-related proteins (SFRPs), a family of tumor suppressor candidate genes, act as Wnt antagonists in the Wnt signaling pathway [65]. SFRP4 is a member of the SFRP family of proteins. SFRP4-dependent Wnt signaling modulation has been reported to be essential for bone remodeling [66]. The sequence of gene13546 was analyzed with ORF Finder [67] and other online softwares [68,69]. Bioinformatics analysis showed that the ORF of *C. elaphus kansuensis* SFRP4 was 624 bp, encoding 207 amino acids. The molecular weight of *C. elaphus kansuensis* SFRP4 protein was 23.37 Kda. The theoretical isoelectric point (pI) was 6.74. The SFRP4 protein contains a conserved domain, CRD_FZ, belonging to the CRD_FZ superfamily.

The higher expression of gene13546 and lower expression of protein13546 in the rapid growth stage indicate that certain factors inhibit the translation of gene13546, which in turn activates the Wnt signaling pathway and promotes the rapid growth of the velvet antler. In the ossification stage, this inhibitory effect is relieved, protein13546 protein accumulates and inhibits the activity of the Wnt signaling pathway, and the growth rate of velvet antler slows down and gradually ossifies. This may explain the timely cessation of deer antlers during proliferation without unlimited growth or cancerization.

GO and KEGG annotations provide useful resources for the further identification of specific cellular structures, pathways, processes, and protein functions in antlers. Our data revealed that many DEGs and DEPs in the cartilage tissue of antlers at different growth stages. Thus, the genes involved in cancer, angiogenesis, and chondrogenesis may play key roles in antler development during the rapid growth stage.

## 5. Conclusions

The selected 488 DEGs and 16 DEPs that had differential expression levels at 30 d vs. 60 d and 60 d vs. 90 d but not at 30 d vs. 90 d may have research potential in regeneration of velvet antler. The gene13546 and its coding protein protein13546 annotated in the Wnt signaling pathway may possess important biological functions in rapid antler growth.

## Figures and Tables

**Table 1:** Top unigenes with highest expression in three libraries.

d30		d60		d90	
Gene ID	FPKM	Gene ID	FPKM	Gene ID	FPKM
gene4406	21762.164	gene4406	22433.19609	gene4406	18476.40401
gene13363	9940.114655	gene13363	17511.18606	gene13363	13310.13206
gene7568	6636.798024	gene7568	7031.922373	gene1629	9726.051422
gene2851	3166.407348	gene21360	3111.317734	gene7568	8106.755774
gene21360	3036.281532	gene2851	3085.655884	gene21360	3165.661509
gene7842	2921.125488	gene2852	2773.071777	gene2851	2506.976623
gene7323	2801.431152	gene7323	2708.365234	gene17381	2460.772705
gene2852	2557.674316	gene17381	2611.812988	gene7842	2424.758789
gene7381	2540.665881	gene10381	2538.190388	gene2852	2174.088867
gene10381	2413.023017	gene7842	2481.294678	gene7323	2160.519531
gene19732	2199.416988	gene1629	2477.907784	gene3336	2003.781029
gene3336	2107.003662	gene9487	2229.203369	gene10381	1976.832594
gene16931	2031.898727	gene3336	2209.905213	gene19732	1887.025254
gene17381	2001.166626	gene15888	2127.262207	newGene_15708	1807.805496
gene9487	1994.406006	gene18121	2125.092085	newGene_29845	1741.988892
gene22181	1956.759766	gene19732	1942.814499	newGene_2805	1664.798706
gene18121	1939.410617	gene3351	1925.796814	gene3351	1636.892456
gene723	1862.862183	gene16931	1909.572655	gene15888	1556.295532
newGene_2805	1826.791504	gene13127	1883.147461	gene16931	1527.461914
gene15888	1821.269531	gene18131	1821.30127	gene723	1490.671143

**Table 2:** Top proteins with highest expression in three libraries.

d30		d60		d90	
Protein ID	Expression	Protein ID	Expression	Protein ID	Expression
protein7361	1.686	protein1174	1.719	protein3419	3.398
protein14704	1.666	protein19035	1.691	protein17593	3.243
protein1519	1.664	protein11670	1.6	protein16793	3.082
protein4597	1.66	protein10984	1.596	protein9356	2.86
protein4576	1.648	protein1517	1.5	protein10194	2.786
protein1521	1.635	protein11698	1.496	protein11790	2.785
protein11686	1.592	protein13127	1.464	protein4470	2.669
protein2427	1.543	protein457	1.425	protein6646	2.177
protein1512	1.497	protein10013	1.417	protein8135	2.087
protein15147	1.443	protein14729	1.416	protein10206	2.007
protein4602	1.412	protein16576	1.405	newProtein_14627.3	1.908
protein1875	1.389	protein11169	1.403	protein1493	1.899
newProtein_6084.1	1.386	protein2538	1.402	protein3325	1.867
protein3788	1.371	protein13191	1.401	protein8462	1.828
protein4604	1.369	protein10014	1.393	protein2690	1.827
protein1514	1.367	protein1982	1.383	protein16950	1.805
protein629	1.353	protein7765	1.362	protein6800	1.775
newProtein_16623.2	1.348	protein14730	1.359	protein13164	1.68
newProtein_20523.1	1.348	protein7142	1.354	protein6020	1.66
protein15292	1.345	protein5871	1.352	protein16569	1.66

**Table 3:** Integrative analysis of transcriptome and proteome.

		d30 vs. d60					d30 vs. d90					d60 vs. d90			
		mRNA		Protein			mRNA		Protein			mRNA		Protein	
	Total	Up	Down	Up	Down	Total	Up	Down	Up	Down	Total	Up	Down	Up	Down
NDEPs_DEGs	76	44	32	0	0	76	36	40	0	0	80	18	52	0	0
DEPs_NDEGs	24	0	0	11	13	213	0	0	117	96	257	0	0	125	132
DEPs_DEGs	8	5	3	3	5	25	10	15	16	9	45	13	32	30	15
DEPs_DEGs same tend	0	0	0	0	0	9	5	4	5	4	16	7	9	7	9
DEPs_DEGs reverse tend	8	5	3	3	5	16	5	11	11	5	29	6	23	23	6

**Abbreviations:** DEGs = differentially expressed genes; DEPs = differentially expressed proteins; NDEPs, non-differentially expressed proteins; NDEGs, non-differentially expressed genes.

## Data Availability

The transcriptomes generated and analyzed in this study are available in the NCBI SRA database under BioProject Accession Numbers PRJNA772802 (Transcriptome profiles of velvet antler in Gansu red deer. Available online: http://www.ncbi.nlm.nih.gov/bioproject/772802 (accessed on 19 March 2022)). Readers can access the proteome data, supporting the conclusions of the study by requesting them from the authors. The mass spectrometry proteomics data have been deposited to the ProteomeXchange Consortium (http://www.proteomexchange.org (accessed on 19 October 2021)) with the dataset identifier PXD032668.

## Supplementary Materials

The following supporting information can be downloaded at: https://www.mdpi.com/article/10.3390/ani12070934/s1, Figure S1. KEGG classification of all DEGs; Figure S2. GO classification of DEGs in 30 d vs. 60 d, 30 d vs. 90 d, and 60 d vs. 90 d; Figure S3. KEGG classification of DEGs in 30 d vs. 60 d, 30 d vs. 90 d, and 60 d vs. 90 d; Figure S4. GO classification of DEPs in 30 d vs. 60 d, 30 d vs. 90 d, and 60 d vs. 90 d; Figure S5. KEGG classification of DEPs in 30 d vs. 60 d, 30 d vs. 90 d, and 60 d vs. 90 d.
