# Supplementary material for: Integrative Analyses of Antler Cartilage Transcriptome and Proteome of Gansu Red Deer (Cervus elaphus kansuensis) at Different Growth Stages
Source: Animals (Basel). 2022 Apr 6;12(7):934. doi: 10.3390/ani12070934 (PMC8997108; doi:10.3390/ani12070934)
Supplement: Supplementary file 1 [file animals-12-00934-s001.zip › Supplementary figures.pdf]

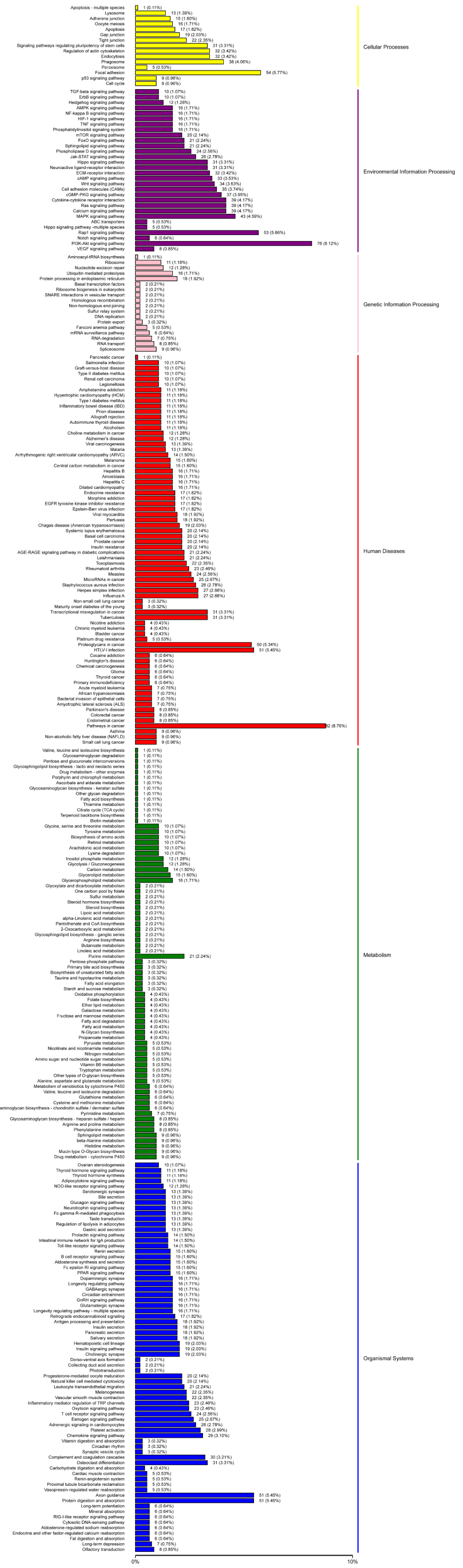
Cellular Processes
Environmental Information Processing
Genetic Information Processing
Human Diseases
Metabolism
Organismal Systems
0%
10%

Figure S1. KEGG classification of all DEGs. For KEGG annotation of all DEGs, the ordinate indicates the name of pathways, and the abscissa is the number and percentage of DEGs classified to the pathways. DEGs were mainly classified in “Pathway in cancer”, “P13K-Akt signaling pathway”, “Focal adhe-sion” and “Rap1 signaling pathway”.

A

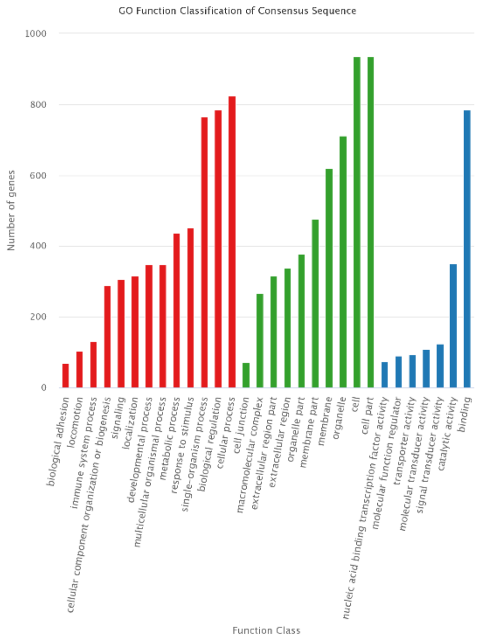

d30 vs. d60

B

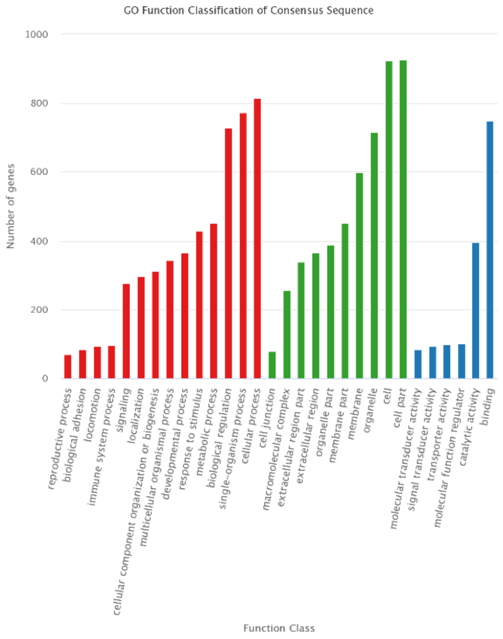

d30 vs. d90

C

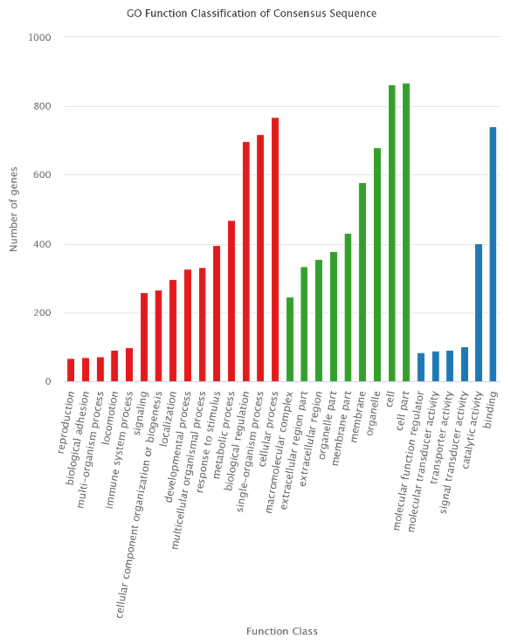

d60 vs. d90

Figure S2. GO classification of DEGs in 30d vs 60d, 30d vs 90d and 60d vs 90d. For GO annotation, the abscissa is the GO terms and the ordinate indicates the number of DEGs annotated to GO terms. (**A)** GO classification of DEGs in comparation of 30d vs 60d. (**B)** GO classification of DEGs in comparation of 30d vs 90d. (**C)** GO classification of DEGs in comparation of 60d vs 90d.

A

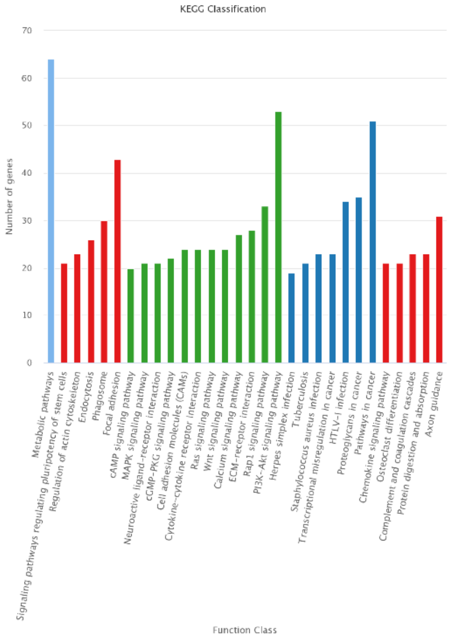

d30 vs. d60

B

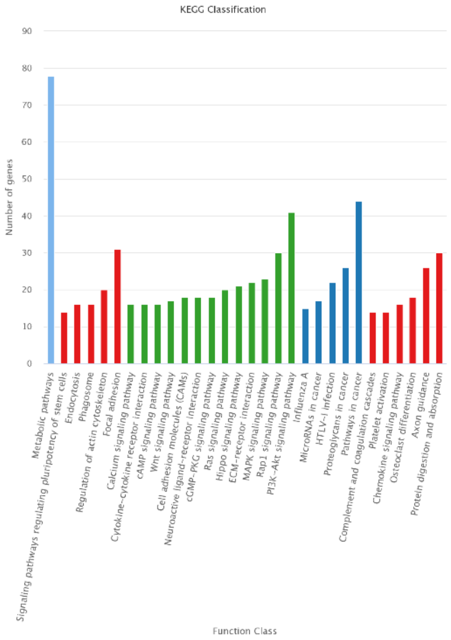

d30 vs. d90

C

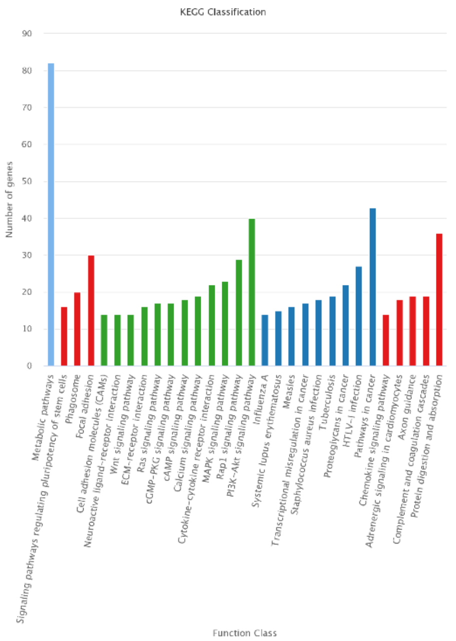

d60 vs. d90

Figure S3. KEGG classification of DEGs in d30 vs d60, d30 vs d90 and d60 vs d90. For KEGG annotation, the ordinate indicates the name of pathways, and the abscissa is the number and percentage of DEGs classified to the pathways. (**A**) KEGG classification of DEGs in comparation of 30d vs 60d. (**B**) KEGG classification of DEGs in comparation of 30d vs 90d. (**C**) KEGG classification of DEGs in comparation of 60d vs 90d.

A

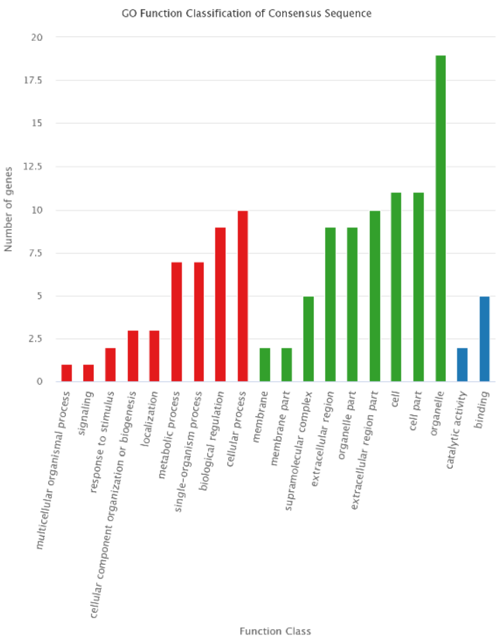

d30 vs. d60

B

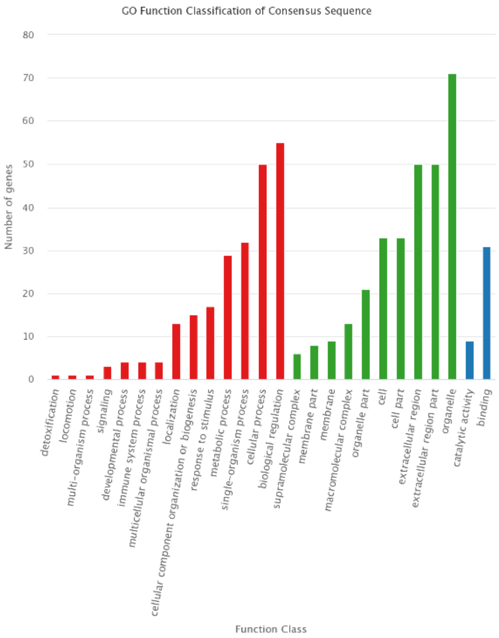

d30 vs. d90

C

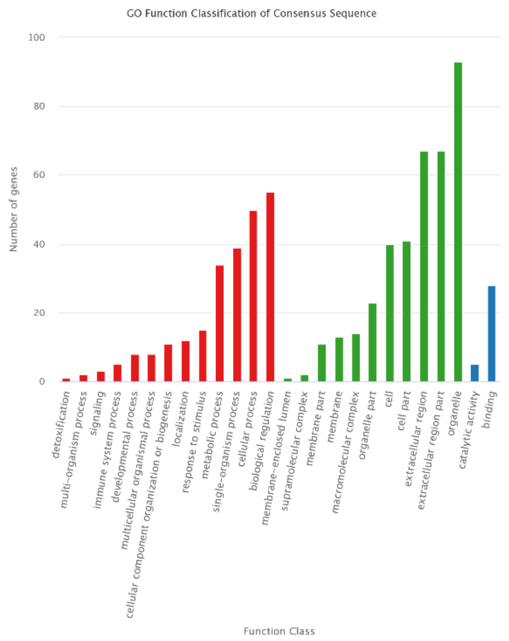

d60 vs. d90

Figure S4. GO classification of DEPs in d30 vs d60, d30 vs d90 and d60 vs d90. For GO annotation, the abscissa is the GO terms and the ordinate indicates the number of DEPs annotated to GO terms. (**A**) GO classification of DEPs in comparation of 30d vs 60d. (**B**) GO classification of DEPs in comparation of 30d vs 90d. (**C**) GO classification of DEPs in comparation of 60d vs 90d.

A

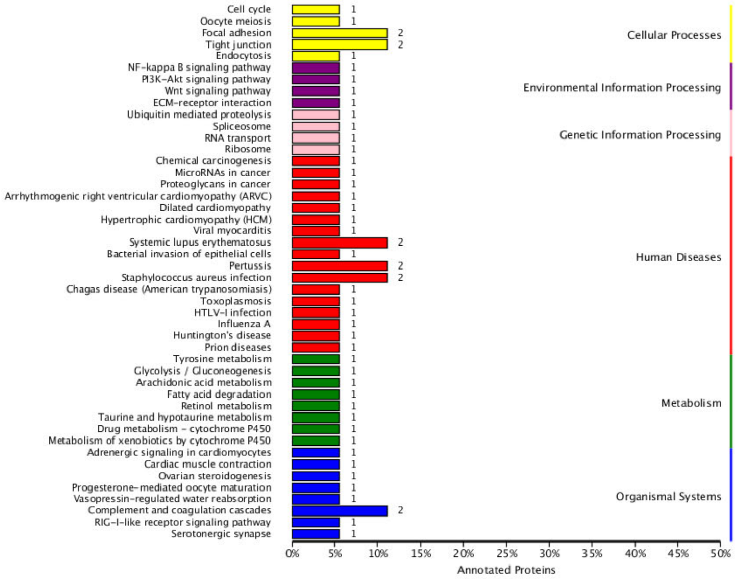

d30 vs. d60

B

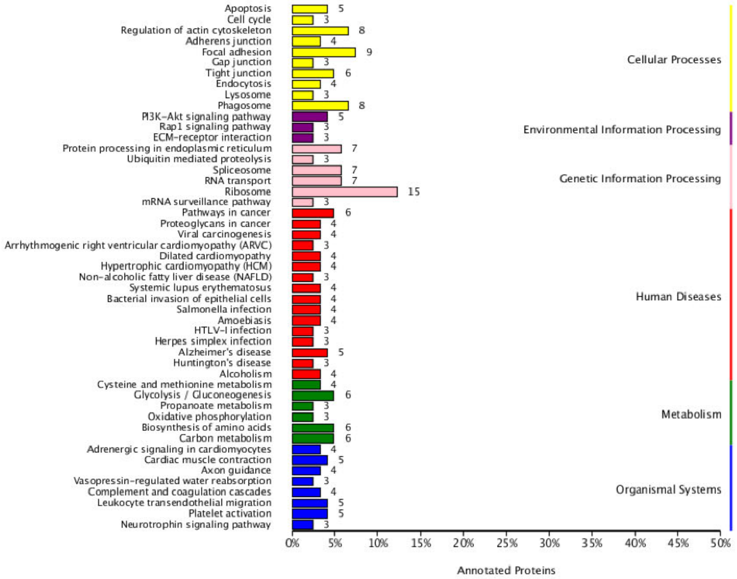

d30 vs. d90

C

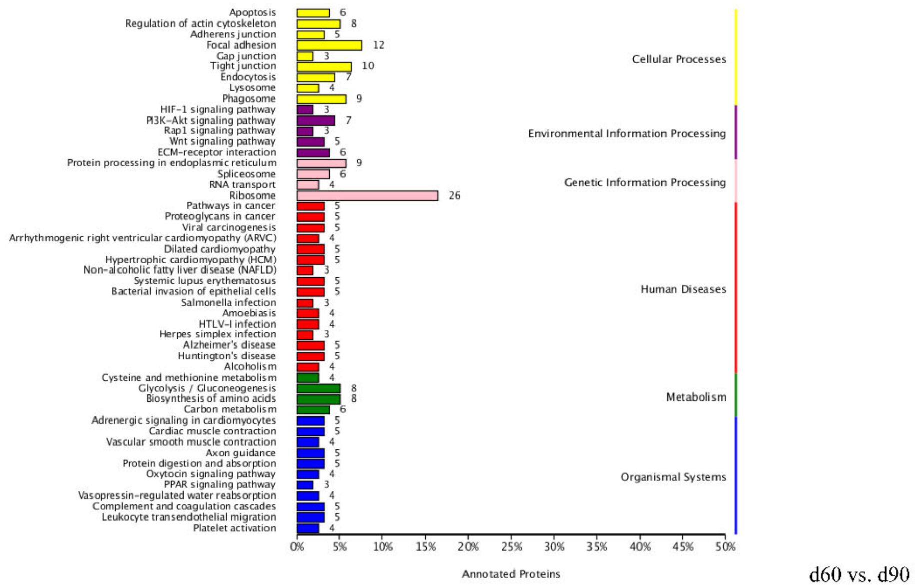

Figure S5. KEGG classification of DEPs in d30 vs d60, d30 vs d90 and d60 vs d90. For KEGG annotation, the ordinate indicates the name of pathways, and the abscissa is the number and percentage of DEPs classified to the pathways. (**A**) KEGG classification of DEPs in comparation of 30d vs 60d. (**B**) KEGG classification of DEPs in comparation of 30d vs 90d. (**C**) KEGG classification of DEPs in comparation of 60d vs 90d.
